# Social Determinants of Health Assessed Among Nurses: A KAP-Oriented Systematic Review Using the Dahlgren-Whitehead Rainbow Model

**DOI:** 10.3390/healthcare14050560

**Published:** 2026-02-24

**Authors:** Alessandra Improta, Erika Renzi, Nicolò Panattoni, Maila Ruggeri, Marco Di Muzio, Maurizio Marceca, Fabio Fabbian, Azzurra Massimi, Emanuele Di Simone

**Affiliations:** 1 Department of Biomedicine and Prevention, University of Rome Tor Vergata, 00133 Roma, Italy; improtalessandra@gmail.com; 2 Department of Public Health and Infectious Diseases, Sapienza University of Rome, 00185 Rome, Italy; erika.renzi@uniroma1.it (E.R.); maurizio.marceca@uniroma1.it (M.M.); azzurra.massimi@uniroma1.it (A.M.); 3 Nursing Research Unit IFO, IRCCS Regina Elena National Cancer Institute, 00144 Rome, Italy; 4 University Hospital ‘Policlinico Umberto I’, Sapienza University, 00185 Rome, Italy; m.ruggeri@policlinicoumberto1.it; 5 Department of Wellbeing, Health and Environmental Sustainability, Sapienza University of Rome, 00185 Rome, Italy; marco.dimuzio@uniroma1.it; 6 Faculty of Medicine, Pharmacy and Prevention, University of Ferrara, 44121 Ferrara, Italy; f.fabbian@ospfe.it; 7 Department of Medical, Movement and Wellbeing Sciences, University of Naples ‘Parthenope’, 80133 Naples, Italy; emanuele.disimone@uniparthenope.it

**Keywords:** social determinants of health, health equity, knowledge, advocacy for health, attitude, practice, nurses, nursing students, systematic review

## Abstract

**Highlights:**

**What are the main findings?**

- The SDoH that are most frequently explored in relation to nurses’ and students’ knowledge, attitudes and practices are poverty, social justice, social gradient, social inclusion and exclusion, discrimination, diversity, equity and inequality, food insecurity and access to nutritious food, employment status, geographical isolation, healthcare services, housing difficulties, transportation, social support, individual lifestyle factors, and health literacy.
- The SDoH needing further focus are those linked to stable individual conditions, like age, sex, and constitutional factors, as these did not arise in connection with nurses’ or students’ knowledge, attitude and practice.

**What are the implications of the main findings?**

- Mapping the most and least frequently assessed SDoH in relation to knowledge, attitudes and practices can inform the development of nursing education and training.
- Promoting health and health equity requires addressing SDoH through collaborative action across sectors. Nurses can be agents of change in this process.

**Abstract:**

**Background and Objectives**: Social Determinants of Health (SDoH) are factors that can contribute to health inequities. Improving the conditions in which people are born, grow, and live requires collaboration between professionals from different health sectors. Given their health and well-being-focused care, nurses are crucial to promoting health equity in the care they provide. Thus, their knowledge, attitudes, and actions—i.e., practice—(KAP) regarding SDoH could serve as a helpful starting point for promoting care that also focuses on non-medical factors. This study aims to map the SDoH assessed in the literature in relation to nurses’ and nursing students’ KAPs, using the Dahlgren–Whitehead Rainbow Model as a logical framework. **Methods**: Following PRISMA guidelines, a systematic literature review was conducted using PubMed, Scopus, Web of Science, CINAHL, and PsycINFO. Records published until June 2024 were selected from primary studies involving nurses and nursing students, with no time limits. The assessed determinants were adapted and categorised according to the Rainbow Model Levels. **Results**: 22 results were eligible. The SDoH (in general), poverty, social justice, social gradient, social inclusion and exclusion, discrimination, diversity, equity and inequality, food insecurity and access to nutritious food, employment status, geographical isolation, healthcare services, housing difficulties, transportation, social support, individual lifestyle factors, and health literacy were assessed on KAPs. Conversely, health equity has been assessed just for knowledge and attitudes. Considering the latter level of the Rainbow Model and the relative categorisation of the results, age, sex, and constitutional factors were not examined in the studies included in this review. **Conclusions**: This review maps the most and least frequently assessed SDoH in relation to KAP. As nurses are essential to providing care that considers SDoH, improving health outcomes, and addressing health inequities, and advocating for community health, it would be valuable to enhance nursing education from baccalaureate through postgraduate courses. Moreover, a strong relationship with different healthcare professionals is needed.

## 1. Introduction

Nurses are key health professionals in primary healthcare: they are often the first health professionals people meet, are embedded in their communities, share their culture, and can respond effectively to patients’ needs, families, and communities [1]. Nurses, given their professional background, are well-positioned to tailor interventions to reduce health disparities and accommodate the specific needs of populations, thereby fostering health equity [2]. Nursing is founded on a global view of practice that considers the patient’s environment and lifestyle to identify physical, mental, and social health risks. In this way, recent reforms, such as the one in Italy that facilitates access to primary care for socially vulnerable people, including the homeless, provide nurses, in collaboration with other professionals, the opportunity to demonstrate their competence in promoting health and equity [3].

Health equity aims to achieve the highest health standards for everyone, with a focus on those at risk due to social conditions [4], and is achieved when everyone can reach their full health potential [5]. Conversely, health disparities are defined as avoidable variations in disease burden, injury, violence, or the chances of reaching optimal health, experienced by socially disadvantaged racial, ethnic, and other demographic groups and communities [6,7]. Health equity requires addressing health disparities: *reducing disparities indicates progress toward health equity*, achieved by improving the health of disadvantaged groups, not by worsening the health of advantaged ones [4,7]. Addressing the Social Determinants of Health (SDoH) is the key to reducing health inequalities and promoting health equity across the population [8,9]. The general population does not clearly understand the SDoH [10], which represent all non-medical factors that influence health outcomes, as demonstrated by a worldwide Google Trends analysis, showed high search volumes for generic terms such as “social support” (Relative Search Volumes 59.2 ± 11.8) but very low interest in more specific SDoH-related constructs such as “financial toxicity” (Relative Search Volumes 4.8 ± 4.7) [10]. The SDoH cover the conditions in which people are born, grow up, work, live and grow old, as well as the broader set of forces and systems that shape daily life [11]. Understanding their impact and, subsequently, their importance is essential to addressing each level involved (from individuals to communities), and, after more than 30 years, the Rainbow Model remains one of the most effective approaches to their comprehension, as it encourages multisectoral action, avoiding a medical approach to non-health issues, and promotes shared responsibility for health across different sectors; differently, other models, such as Diderichsen’s model, focus primarily on explaining health inequalities by looking at how individuals’ living conditions (due to social position) generate them [12,13]. It emphasises that the SDoH affect health at multiple levels, ranging from societal influences, including general socioeconomic, cultural, and environmental conditions, to more specific factors such as living and working conditions, and ultimately to the individual level [14]. Basically, the SDoH help to understand how materialist and structuralist health inequities (referring respectively on material circumstances and societal/institutional systems, manifest in systematic, avoidable, unfair and unjust differences in health and well-being outcomes [15,16,17]), persist throughout life and explain, for example, how the health and longevity of women differ from men due to the SDoH [18]. In this context, nurses play a central role in advancing health equity by screening for the SDoH, collaborating with community partners to address social needs, and advocating for systemic changes, working on upstream factors influencing health [19,20,21]. The SDoH are integrated into nurses’ care, but they can at times pose challenges for nurses [22,23]. Promoting health and well-being for all should be a priority that requires a collective and sustained commitment, and nurses should be fully supported with robust education, training, resources, and autonomy [24]. Indeed, historically, nursing curricula have emphasised clinical skills, whereas topics related to social justice and equity have been left to community health courses [25]. Given the different perspectives previously explored, this study aims to map which SDoH have been assessed in the literature in relation to the knowledge, attitudes and practice (KAP) of nurses and/or nursing students, considering that the KAP framework could represent a helpful way to find out training or educational needs [26].

Knowledge is defined as the ability to acquire and use information, attitudes are considered the tendency to see, interpret, and organise opinions, and practice is intended as the application of rules and knowledge leading to action [27]. Finally, to excel in their essential role, nurses need to be equipped with awareness, comprehensive knowledge, and the skills and abilities to effectively address or integrate SDoH into their daily activities, thereby contributing to reducing inequalities and promoting health equity.

## 2. Materials and Methods

A systematic literature review was undertaken in accordance with the updated Preferred Reporting Items for Systematic Reviews and Meta-Analyses (PRISMA) guidelines [28]. For reporting, we followed the Preferred Reporting Items for Systematic Reviews and Meta-Analyses checklist obtained from the Equator Network (Supplementary File S1). Inclusion and exclusion criteria, information sources, and data collection procedures were defined a priori. The records were managed using bibliographic software (Rayyan—free web and mobile app—and Zotero software—v. 6.0.36). Review protocol has been registered on the Open Science Framework (OSF) platform with the following doi: 10.17605/OSF.IO/5AGQM. Since the review protocol did not involve the collection of primary data, it was not submitted to any Ethics Committee for approval, and informed consent was not required.

### 2.1. Search Strategy

To better define the search strategy, according to the “PEO” approach [29], nurses and nursing students were identified as referee *Populations*, involved in primary studies or academic/professional courses (*Exposition*), and assessed for knowledge, attitudes or practices related to the SDoH (*Outcome*).

A systematic search was conducted in PubMed, Scopus, Web of Science, Cumulative Index of Nursing and Allied Health Literature (CINAHL), and PsycINFO among articles published until June 2024 (the time at which the search was performed). No lower date limit was applied in the collection strategy; therefore, studies were selected solely based on the predefined inclusion and exclusion criteria, regardless of publication year, provided they were indexed through June 2024. Specific search strategies for each database were followed. Furthermore, a comprehensive hand search was conducted to identify additional studies not found during the preliminary research stages.

Regarding the terms, *health knowledge*, *attitudes*, *practice*, *attitude of health personnel*, *health behaviour*, *KAP*, *nurse*, *nursing students* and *social determinants of health* were the keywords identified to construct the search string used in databases. Furthermore, the search terms were mainly searched in the title and abstract. A full description of the search strategy is shown in Supplementary File S2.

All studies with the following characteristics were included: (i) primary studies with nurses and nursing students involved; (ii) studies regarding knowledge, attitudes and practices related to the SDoH; (iii) studies written only in the English language. Articles not available in full-text, grey literature, reviews, or studies focused exclusively on other students or health professionals from other disciplines were excluded. As previously reported, no time limits were applied.

### 2.2. Data Extraction and Synthesis

Each identified record was collected and independently evaluated by two researchers (A.I., E.D.S.). Any doubts during the screening or inclusion phases were resolved by involving a third researcher (E.R.). For each included record, the following information was collected: first author and year of publication; title; country; design/methodology; outcome; sample; main results; SDoH; KAP; and approaches (teaching strategy or instrument(s)). Data collection was performed using an Excel sheet.

#### Framework: Adaptation of the Dahlgren–Whitehead Model

The SDoH that were assessed and investigated in the studies included in this review were categorised using the Dahlgren–Whitehead Model, after adaptation [14,30]. This model categorises the factors that influence health into five levels, as shown below.

The top level of our adapted model, *general socioeconomic*, *cultural*, *and environmental conditions*, addresses broad conditions that shape health inequalities, and it includes crime, violence, equity, gender (as differences tied to societal roles [31]), social exclusion, and conditions that inhibit full societal participation economically, socially, culturally, and politically [32]. We relate this to civic participation, involving various activities benefiting society or the group members [33], and other social phenomena, such as discrimination (favouring dominant groups [34], marginalisation (the process through which persons are peripheralized based on their identities, associations, experiences, and environment [35]), stigma, poverty, social justice (that involves the full participation of all citizens in society and the equal distribution of the relative benefits and burdens [36]), social gradient (the decrease in health status resulting from the decreasing social position [11]), and social statuses, like immigration. We also covered cultural humility and cultural safety as key elements in healthcare: the former drives culturally safe care provided by healthcare professionals through a continuous examination of their values and assumptions, while the latter ensures congruence between a patient’s culture and services provided, by creating an environment in which people feel culturally respected and is a goal for health systems [37]. Given the previously considered factors, we also included cultural and ethnic diversity, race, and spirituality. At this level, spirituality is included because, in reevaluating public health’s future and strengthening it through person- and community-centred approaches, spirituality should be incorporated into the SDoH, valued by individuals and communities, and influence social relationships and civic engagement [38,39,40].

Subsequently, the level targets the material and organisational contexts of everyday life. In details, in *living and working conditions* level, we considered food insecurity, education, employment status (unemployment, job security, income, financial strain), environmental health (including physical conditions and environmental issues like air, water, and soil quality), geographic isolation, healthcare services (disparities, access, availability, and quality), housing difficulties (including homelessness), utilities, and transportation needs.

The third layer, *social and community networks*, covers social support and relationships. The focus is on community cohesion, local support structures, community initiatives, and policies that strengthen mutual support among people and protect them from health hazards [14].

We included lifestyle-related factors and/or habits such as drinking, smoking, addiction and health literacy in the subsequent level *individual lifestyle behaviours* because of their association with health-related behaviours [41,42].

The latter layer comprises age, sex, and constitutional factors (individual-level stable conditions), all personal, immutable characteristics of individuals (e.g., genetic) [14]. We considered the term sex to be the biological differences between females and males [31].

## 3. Results

In total, 1240 records were collected. After removing duplicates (319) and screening titles and abstracts (921), 31 studies were assessed for eligibility. Two studies retrieved after the integrative manual search were assessed for eligibility. Then, 11 were excluded for the following reasons: two were not primary studies, six did not provide any analysis, explanation or assessment of KAP in relation to the SDoH, one did not survey nurses or students, and two did not focus on the SDoH. After full-text evaluation, only 22 papers were included in this review. The overall selection process is shown in the Preferred Reporting Items for Systematic Review and Meta-Analyses (PRISMA) flowchart [28] (Supplementary File S3).

### 3.1. Description of the Included Studies

A summary of the studies included, published between 2009 and 2024, is presented in Supplementary File S4.

Most of the included studies were conducted in the United States (US) (sixteen studies) [43,44,45,46,47,48,49,50,51,52,53,54,55,56,57,58]. Of the other included studies, three were from Canada [59,60,61], one from Australia [62], one from Iran [63] and one was an international study, sampling in both the United Kingdom (UK) and the US [64].

The sample sizes ranged from 7 to 936 participants. Four studies did not report the exact sample size [43,49,61,63].

Two studies assessed KAPs towards the SDoH through focus groups [54,59], one used interviews [60], one used self-report surveys and personal reflections [44], nine used questionnaires [45,47,48,51,52,56,57,63,64], one used a photograph with an essay [62], and one used qualitative interviews and surveys [53]. Five studies used simulation or role-play case studies to assess students’ or nurses’ knowledge, attitudes and practice towards the SDoH [46,50,51,55,58]. Three of the included records describe implemented programs [43,49,61].

Finally, all SDoH assessed and investigated in the included studies were, after adaptation, categorised within the framework of the Dahlgren–Whitehead Model [14,30], as shown in Table 1.

### 3.2. Identified Social Determinants of Health

The SDoH, in general, have been studied three times within our literature [43,44,50], and one explored SDoH universal screening [53]. Three studies also focused on health equity [50,61,63].

At the level of *general socio-economic, cultural and environmental conditions*, poverty is the most studied SDoH (9 studies out of 22) [43,47,49,51,54,55,58,63,64], followed by equity (five studies) [43,46,56,57,59], social justice (4 studies out of 22) [43,56,59,64], diversity (including cultural and ethnic diversity and race) was examined in six studies [43,46,49,50,57,63] and discrimination in four studies [45,50,52,55]. Three studies evaluated social status (e.g., immigration status) [43,50,63] and two studies considered crime and violence [52,61].

Less explored in this level are: cultural humility [49], marginalisation [63], social exclusion [48] and inclusion [57], cultural safety [61], stigma [61], spirituality [49], social gradient [48], civic participation [52], and gender and gender identity [50,63].

Regarding *living and working conditions levels*, the most researched aspect is the one related to healthcare services (in 10 studies out of 22): health service access [43,45,52,61], health disparities [44,45,46,47,59], quality of healthcare [45], healthcare barriers [54], availability of health service [62].

Then, four studies explored nurses’ or students’ KAP related to food insecurity [48,53,60,63] and just one explored aspects related to access to nutritious food [52]. Two studies examined the educational level [50,52]. Indicators related to employment status were examined in five studies evaluating financial strain [53], income [48,52,61,62], unemployment [48], and job security [48].

Only two studies [62,63] looked at indicators related to a healthy environment (e.g., overflowing rubbish bins and filthy toilets or environmental problems related to air, water and soil), just one study looked at utilities [52], and one study investigated geographic isolation [60]. Housing difficulties were examined in seven studies [47,52,53,59,60,61,62]. Next, three studies focused on transportation (as transportation needs and free transportation for health services) [48,52,53].

In terms of *social and community networks*, only three studies (out of 22) have explored aspects of social networks as social support [48,52] and social connectedness [53].

SDoH in relation to *individual lifestyle behaviour* were examined in six studies: three studies examined individual lifestyle factors such as drinking or smoking addiction [48,60,62] and three examined health literacy levels [43,52,53].

Finally, none of the included studies examined SDoH in relation to *individual level-stable conditions* such as age, sex, or constitutional factors.

#### 3.2.1. Knowledge

Fifteen studies investigated nurses’ or students’ knowledge regarding the SDoH and/or health equity [43,44,46,47,48,50,52,55,56,57,58,59,60,61,62].

Nurses or nursing students have been assessed for their knowledge regarding health equity [50,61], SDoH in general [44,50], or specific ones, listed below according to the adopted model:

- *General socioeconomic, cultural and environmental conditions*—poverty [43,47,55,58], social justice [56,59], equity and inequities [46,57,59], social status (immigration status) [43], diversity and cultural diversity [43,57], social gradient [48], social exclusion and inclusion [48,57], crime and violence [52,61], cultural safety [61], stigma [61], discrimination [55];
- *Living and working conditions*—healthcare disparities [44], health disparities [46,47,59], healthcare access [43], availability of health service [62], health service access [52], housing instability [47,52,59,60,62], geographic isolation [60], food insecurity and access to nutritious food [48,52,60], employment status (e.g., unemployment, job security, income) [48,52,62], healthy environment (e.g., overflowing rubbish bins and filthy toilets) [62], transportation [48], educational level [52], utilities [52];
- *Individual lifestyle behaviours*—health literacy [43,52], individual lifestyle factors (e.g., drinking, smoking, addiction) [48,60,62];
- *Social and community networks*—social support [48].

One study examined knowledge of nursing roles in addressing the SDoH and health equity [50].

As well as happening in the general population [10], one study revealed a general lack of knowledge or understanding regarding SDoH [60]. Two studies reported low levels of knowledge to discuss specific SDoH, such as social gradient, food insecurity, social exclusion, unemployment and job security [48] or others such as utilities, income, crime, violence, or housing [52].

Differently, high levels of knowledge have been reported in discussing social support, addiction, and transportation [48], health service access, access to nutritious foods, health literacy, and level of education [52].

The included studies’ participants, engaging in interprofessional experiences [43,44], simulations [46,50,55,58], and specifically designed courses [47,56,59,61], improved their knowledge regarding health equity [61], SDoH in general [44], or specific ones, such as: healthcare disparities [44], health disparities [46], equity and inclusion [57], stigma [61], violence [61], problems related to poverty [47] and related daily challenges, such as discrimination and the lack of opportunities [55], social justice [56], impact of resource scarcity on health and well-being [43].

Participants learnt specific nursing roles in assessing or addressing SDoH [46,50,58,59], social justice, health disparities [59] and achieving health equity [50].

Nurses’ or students’ knowledge has also been assessed through surveys [44,48,50,52,56,57] and interviews [59,60].

#### 3.2.2. Attitudes

Sixteen studies explored nurses’ or students’ attitudes towards health equity and/or SDoH [43,46,47,48,49,50,51,52,53,56,57,58,60,62,63,64].

Nurses or students had been researched for their attitudes towards health equity [63] and such SDoH, as presented below:

- *General socioeconomic, cultural and environmental conditions*—cultural and ethnic diversity (and race) [43,46,49,50,57,63], social gradient [48], social exclusion and inclusion [48,57], poverty [47,49,51,58,63,64], social justice [56,64], spirituality [49], cultural humility [49], discrimination [50], social status (e.g., immigration status) [50,63] civic participation [52], crime and violence [52], equity [57], gender and gender identity [50,63] marginalisation [63];
- *Living and working conditions*—geographic isolation [60], housing instability [47,60,62], healthcare access [43], health disparities [47], availability of health service [62], food insecurity [48,60,63], employment status (unemployment, job security, income) [48,52,62], educational level [50], health service access [52], utilities [52], transportation [48,52], healthy environment [62,63];
- *Social and community networks*—social support [48,52];
- *Individual lifestyle behaviours*—individual lifestyle factors (e.g., drinking, smoking, or other addiction) [48,60,62], health literacy [52].

Participants in studies demonstrated positive attitudes toward SDoH universal screening [53] and toward social justice and poverty [64]. According to Phillips et al., nurses’ confidence varied across different SDoH: study participants reported confidence in discussing access to health services, transport, health literacy and social support, and less confidence in discussing income, civic participation, crime, utilities and violence [52]. Participants in one study found it challenging to care for patients with specific social difficulties, such as homelessness [60]. Students seemed to have positive attitudes towards SDoH and their inclusion in training [63].

Participation in simulations [46,50,51,58], specifically designed courses [47,56], international programs [49] or interprofessional experiences [43], fostered non-judgmental attitudes towards specific SDoH, such as poverty [47] positive attitudes towards marginalised groups [50], a structural explanation of poverty as a social and systemic result [51], confidence in addressing diverse populations’ complex needs [43], and awareness of cultural humility, social inequalities [49], awareness of cultural and ethnic diversity [46], social justice [56] and SDoH in general [49,58].

Nurses’ or students’ attitudes toward SDoH have been assessed using questionnaires and surveys [47,48,50,51,52,53,56,63,64], interviews [53,60] and photography with an essay [62].

#### 3.2.3. Practice

Fifteen studies investigated nurses and/or students’ practice related to SDoH [43,45,46,47,48,49,52,53,54,56,57,58,59,60,61].

Nurses or students in their practice experienced SDoH in general [43] and the following ones:

- *General socioeconomic, cultural and environmental conditions*—social justice [43,56,59], equity [43,56,57,59], social gradient [48], social exclusion and inclusion [48,57], poverty [47,54,58], discrimination [45,52], civic participation [52], diversity (socio-cultural aspects) [57];
- *Living and working conditions*—housing instability (and homelessness) [47,53,60,61], geographic isolation [60], quality of healthcare [45], health disparities [45,46,47], food insecurity and access to nutritious food [48,52,53,60], employment status (e. g. financial strain, job security, income, unemployment) [48,52,53,61], health service access [45], transportation [48,53], healthcare access [61], healthcare barriers [54];
- *Social and community network*—social support [48], social connection [53];
- *Individual lifestyle behaviours*—health literacy [53], addictions [48,60].

In two studies, participants reported limited training regarding SDoH in general [60] and in responding to identified social needs [53]. In one study, participants reported having the skills needed to address diversity, equity and inclusion, and they reported valuing diversity, equity and inclusion in nursing [57]. However, it seems that the likelihood of discussing social gradient, social exclusion, unemployment and job security, civic participation, income, discrimination, utilities, and access to nutritious foods and food insecurity is low [48,52].

Through specifically designed courses [47,56,59,61], participants learned to apply social justice and equity in clinical practice [59], enhanced their autonomy and responsibility to address health risks [47], gained skills to assist clients in accessing health, housing, income, and legal services, and make referrals [61], and increased their awareness of social injustice and confidence in achieving social justice [56].

Engaging in simulations [46,58] helped participants identify health inequalities affecting quality of life, assessment of patients’ needs, offer of community-based resources, and education of patients according to their specific socioeconomic, cultural, and environmental conditions [46], and the development of interpersonal and social empathy [58].

Participation in an international program [49] enhanced practice in complex cultural and socioeconomic contexts [49]. Interprofessional experiences [43,54] empowered participants to identify and address SDoH and improve household services [43], while visualising impacts on health outcomes [54].

Nurses’ or students’ practice related to SDoH has been evaluated using surveys [45,48,52,56,57] and interviews [53,54,59,60].

## 4. Discussion

This study aimed to identify SDoH evaluated for nurses and nursing students by the KAP framework [26] and the Rainbow Model [14]. Public health needs to shift from a biomedical to a social-ecological paradigm to address SDoH and promote health equity effectively [65]. Our results show that, in the literature, health equity has never been assessed for practice, but only for knowledge [50,61] and attitude [63]. Among the less explored SDoH, none are related to health services; instead, they pertain primarily to socioeconomic, cultural, and living conditions, as well as to social and community networks. Although positive attitudes towards universal screening for SDoH have been demonstrated [53], nurses sometimes feel challenged to care for patients with special social needs, and they addressed their initial unpreparedness by gaining experiential knowledge and integrating it into their clinical practice [60].

Cultural humility [49] and cultural safety [61] are essential concepts for the provision of high-quality, equitable and inclusive healthcare [66], but are undervalued in relation to nurses’ or students’ KAP, as shown by our findings: none were assessed for practice, only for knowledge [61] or attitude [49]. All health professionals should be trained to recognise patients’ specific cultural needs and to understand the diversity of approaches to healthcare in multicultural societies [67]. Training healthcare providers in cultural humility can address the risks of stereotyping and stigmatisation [68]. Cultural safety recognises the specific needs of minority and marginalised groups concerning their cultural traditions and identities; in this way, structural and systemic barriers that may affect their access to healthcare and the quality of care they receive, including the socio-economic factors that influence health and well-being, can be identified and overcome [69,70]. Among our findings, marginalisation has been assessed only once in relation to attitudes [63]. Integrating marginalisation and SDoH frameworks can improve understanding of health inequalities and inform intervention planning to address the chronic disease burden, thereby improving access to healthcare for vulnerable populations [35]. Social stigma and discrimination worsen health inequalities [71,72]. Nurses are uniquely positioned to mitigate inequalities through interventions that understand and address the unique cultural needs and experiences of minorities, due to their comprehensive view of the individual [71]. This review found that stigma [61] and spirituality [49] are undervalued and studied only regarding attitudes, despite the former having been identified as a key determinant of health and health inequalities due to its impact on access to health-promoting resources and exposure to stress [73], while the latter supports well-being by providing stress and illness coping mechanisms, as seen during the COVID-19 pandemic challenges [74]. Although specific populations, such as immigrants and refugees, experience exclusion from participation in social, economic, civic, and political spheres, our findings suggest that these themes have not been much researched for nurses’ or students’ KAP [48,56,57]. Additionally, civic participation is one of the least researched SDoH (only for attitudes [52]). Health professionals should explicitly address civic participation in their practices, as it may be an important pathway to health equity rather than a purely political or social issue: better health enables civic participation, and civic engagement, in turn, positively influences health outcomes [75]. A primary way civic participation enhances health is by building social capital (networks, norms, and social trust that encourage cooperation for mutual benefit) [33]. This, in turn, raises awareness of opportunities for physical activity and fosters a stronger sense of purpose [33]. So, in the primary care context, professionals can strengthen social connectedness, enhance self-efficacy (people’s belief in their ability to act), and encourage collective action (to improve shared conditions) [75]. For instance, encouraging civic participation by promoting youth involvement in community service and public health media campaigns is essential for fostering lifelong engagement and emphasising civic participation as key SDoH [33]. In this way, primary care not only treats illness but also helps individuals and communities gain agency and power, thereby supporting health equity.

Notably, determinants such as social connectedness [53] received scarce attention, as emerged from findings: just social support has been assessed twice for attitudes [48,52]. It is not surprising that data on nurses’ or students’ KAP regarding indicators of social connectedness (e.g., social capital, social support, social isolation, and loneliness) are scarce, as these indicators are often not considered [76,77]. Due to the previous consideration regarding the less explored SDoH, it is important to underline that the concept of social gradient emerged from only one study [48]. Thus, considering the strong link between individuals’ socioeconomic conditions and their health outcomes [78], it is necessary to increase professionals’ KAPs towards this important concept in light of addressing SDoH routinely.

Despite the scarcity of literature examining nurses or students’ KAP towards SDoH as geographic isolation [60], utilities [52] and educational level [50,52], it is necessary to explore their management in clinical practice or in public health facilities and services, due to their impact on overall health [79,80]. Moreover, low educational attainment, a key upstream SDoH factor, is linked to worse health outcomes, well-being and life expectancy [81,82,83]: adults with less education report more chronic conditions, functional limitations, and worse overall health, and their educational attainment also influences their children’s health [81].

None of the included studies considered individual factors such as age, sex and other constitutional factors. This is a significant gap given several phenomena such as ageism, which encompasses stereotypes, prejudice and discrimination against oneself or others based on age [84], resulting in various harmful effects, including age-based health inequities and poorer health outcomes [85]. Furthermore, negative attitudes towards age and ageing also contribute to workforce shortages in elderly care sectors, such as residential care and nursing, leading to a lack of interest in gerontological care and potentially lower quality of care [86].

Moreover, due to the increasing role of digital determinants in health inequities (including disparities in smartphone ownership and digital literacy across income levels and genders) [87,88], Jahnel et al. suggested integrating digital influences into the original Rainbow Model [14], acknowledging that digital transformation affects each level of it, from policy to healthcare access (e.g., telemedicine) [87]. Results indicate that digital factors have not been considered in the literature. Assessing health professionals’ KAPs regarding digital transformation could be valuable for ensuring equitable healthcare access in future research.

Following the examination of the less-researched SDoH, the most extensively evaluated ones in terms of KAP were delineated. On the other hand, among the most assessed SDoH for knowledge, from our results, emerged crime and violence [52,61], which have a substantial impact on health [89]. A disproportionate risk of violence is compounded by inequalities based on race, socio-economic status, gender and geographic location, making some communities at high risk [90]. It is crucial to consider systemic marginalisation, income inequality and other structural factors which stratify communities [91]. Findings also highlight housing and homelessness [47,52,59,60,62], factors that often challenge healthcare professionals due to patients’ unique needs [60] and are associated with increased healthcare utilisation [91]. Despite the fact that knowledge regarding access to healthcare has been the most researched [43,44,46,47,50,52,59,61,62], it represents only 10% of the inequality burden among all the other factors, whilst social and human capital (such as isolation, lack of control, trust in others and low educational attainment), although less explored, accounts for 19%, so nearly twice as much [92]. Furthermore, nurses’ or students’ knowledge assessment of individual lifestyle factors (e.g., addiction, smoking, drinking) [48,60,62] is essential, given their modifiable nature and significant impact on health [89,93].

Among the most assessed SDoH for nurses’ or students’ attitudes, on the level of *general socio-economic, cultural and environmental conditions*, we found social status (i.e., immigration status) [50,63], poverty [47,49,51,58,63,64], gender and gender identity [50,63], diversity (also cultural and ethnic diversity) [43,46,49,57], and race [50,63]. This literature review revealed that there is no explicit reference to racism, just race. Including race, but not racism, as a social determinant of health may lead to the misuse of race terms in explaining health inequalities [94]. It is essential to explore nurses’ and nursing students’ attitudes and perceptions of poverty, as students often adopt more individualistic explanations that emphasise personal responsibility and are less likely to link poverty to health disparities or advocate for policies [51,95]. Given its relevance, it may be helpful to revisit curricula to promote a systemic view of poverty as a cause of health inequities [95]. For instance, discussion on poverty often overlook oppression; the ones regarding race tend to omit racism, and similarly those regarding sex and homosexuality fail to address sexism and homophobia: SDoH should be considered products of structural conditions that require active intervention, moving education from a descriptive model to a training model that is both justice and systems oriented and equips students with the competencies to intervene at individual, organisational and policy levels [96]. Alongside revising curricular content, appropriate teaching strategies (e.g., interactive, immersive experiences or role-play-based simulations) must be implemented. Overall, immersive teaching methods could be beneficial in preparing students to advocate for health equity, enhancing their knowledge, awareness, and skills, and appear to be well-received by students [97,98,99].

In addition, gender identity requires unique considerations when providing comprehensive care to transgender people [100]. Assessing professionals’ attitudes is crucial since nurses, interacting with sexually and gender-diverse people across the lifespan, can contribute to combat stigma, build trust, and advocate for these groups on both the practice and policy levels, and reduce inequalities [101].

Physical environmental conditions significantly impact health and well-being, with disadvantaged groups at higher risk due to unequal exposure to air pollution, inadequate access to clean water and sanitation, and energy insecurity, with low-income households facing significantly higher risks of overcrowding, exposure to polluting fuels, and lack of access to essential resources [102]. Findings on attitudes towards a healthy environment highlight issues like overflowing bins, dirty toilets, air, water, and soil quality [62,63]. This is a relevant result because of the impact of the outdoor environment on health outcomes and life expectancy, which reflects nursing awareness regarding the patients’ living physical environment (e.g., in poor hygienic conditions), conditions that they could use as ‘red flags’ for deeper screening health and social problems, and then consider into care plans, and also with the involvement of other professionals [21,103].

Among the most assessed SDoH for nurses’/students’ practice, emerged social justice [43,55,58], equity (and inequities) [43,56,57,59], discrimination [45,52], food insecurity [48,52,53,60], conditions related to employment [48,52,53,61], transportation needs [48,53] (such as free transportation for health services [48]). In recent years, the economic issues in the healthcare sector, the pandemic challenge, and (inter) community conflicts have exacerbated social and health inequities [104]. Despite this, nursing has historically focused on health equity and social justice, and also through activism, as evidenced by movements such as the *public health nursing movement* and the *nurse practitioner movement*, which provide actions in improving access to care or by introducing new dedicated services, under the equity lens [104]. More in depth, during the COVID-19 pandemic, race/ethnicity, poverty, income level, housing conditions, access to healthcare services, occupation, transportation, education, air quality, and food security emerged as being associated with adverse health outcomes [105]. Nurses must prioritise SDoH alongside other physical aspects of care through a comprehensive assessment of a patient’s social and environmental conditions [21]. Considering the practice of nurses or students, assessing these previously identified SDoH is essential because health inequalities are not merely differences in health; they refer to poorer health outcomes among socially disadvantaged groups, particularly those who face any form of discrimination [4].

Following the mapping of the most and least researched SDoH in the nursing literature, it is essential to emphasise the relationship observed in one of the included studies between the levels of nurses’ knowledge, skills, and attitudes in practice: as knowledge increases, so do skills and positive attitudes [57]. In addition, these findings could serve as a guide for nursing education by highlighting SDoH that need more attention for nurses’ and students’ KAPs. Achieving health equity requires addressing systemic challenges and implicit biases within healthcare; nurses can act as change agents by implementing SDoH screening tools, advocating systemic reform, collaborating with interdisciplinary teams, integrating social care into healthcare delivery, fostering community partnerships, and encouraging interpersonal skills to mitigate bias and enhance patient care [19,21].

### Limitations

We acknowledge the heterogeneity of the findings and the limitations of the results. This review identified a wide range of methodologies for assessing knowledge, attitudes, and behaviours. Given the objective of mapping SDoH assessed in nursing fields, no formal risk-of-bias evaluation of the included studies was performed. Therefore, the work focused on identifying SDoH domains rather than drawing conclusions regarding effects or associations. Then, considering the aims of our study, our findings do not establish an overall trustworthiness of the evidence. In addition, no stratification of the data by the healthcare system (and related cultural differences) or by KAPs before and after interventions (where applicable) was performed. Moreover, studying SDoH and professionals’ KAPs is challenging, and a study focusing solely on nurses or students may be insufficiently comprehensive. The focus of this review was on nurses’ and students’ KAPs; the relationship between nursing and social workers or social services was not considered. We are aware that these two healthcare professionals should communicate closely to evaluate the resources dedicated by different healthcare systems.

In conclusion, readers must be aware that our findings constitute a knowledge and relevance contribution to SDoH for nursing professionals. Further studies could replicate our approach by comparing multiple professions across both the healthcare and social sectors, enriching this mapping by evaluating the overall quality of evidence in SDoH studies and by considering different healthcare systems.

## 5. Conclusions

Determining health inequities, SDoH converge and accumulate throughout life, shaping the health of population groups according to their social status, such as education, ethnicity, gender, gender identity, income, occupation and sexual orientation [15,16,17]. In this context, nurses are strategically positioned to make a significant contribution to achieving health equity [106]. This systematic literature review examined the SDoH assessed in existing research on nurses’ and students’ knowledge, attitudes and practices. Dahlgren-Whitehead’s Rainbow Model [14] provided an invaluable framework for classifying SDoH and their impact on health and well-being. By mapping the most and least frequently assessed SDoH across knowledge, attitudes, and practices, this review uncovers significant potential for transformative improvements in nursing education and training, as evidenced by the interplay among knowledge, skills, and positive attitudes in nursing practice. Addressing the SDoH is central to promoting health and health equity and requires synergistic, collaborative action across sectors [48,106]. Nurses, through their proximity to the population, are in a unique position to advocate for health and promote patient-centred care that accounts for the full context in which patients are born, grow, work, live, and age, thereby promoting health equity, addressing inequalities, and improving patient outcomes. This fundamental action, which requires adequate university training, as well as ethical awareness of the profession, should be exercised not only at an individual level but also as a professional community through the adoption of positions and initiatives by professional nursing associations, ideally in collaboration with other healthcare professional association [2,5,11,16,17,19,20,21,22,23,24,25,30,35,43,44,45,47,48,50,53,55,56,59,60,61,62,63,65,73,76,78,90,102,104,106].

## Figures and Tables

**Table 1:** SDoH classification adapted from Dahlgren–Whitehead Model.

**Health equity**		**K**	**A**	**P**	**Studies**
		x			Phan, 2020 [50]
		x			Daly, 2022 [61]
			x		Mohammadi, 2023 [63]
**SDoH**		**K**	**A**	**P**	**Studies**
*SDoH general evaluation*				x	De Los Santos, 2014 [43]
		x			Addy, 2015 [44]
		x			Phan, 2020 [50]
			x		Kostelanetz, 2022 [53]
**General socioeconomic, cultural and environmental conditions**	*Poverty*	x	x	x	Decker, 2017 [47]
		x			De Los Santos, 2014 [43]
				x	Powers, 2022 [54]
		x	x	x	Holland, 2024 [58]
			x		Mohammadi, 2023 [63]
			x		Kuehn, 2020 [51]
			x		Velez, 2020 [49]
			x		Scheffer, 2019 [64]
		x			Killion, 2022 [55]
	*Social justice*	x		x	Cohen, 2009 [59]
				x	De Los Santos, 2014 [43]
		x	x	x	Crawford, 2022 [56]
			x		Scheffer, 2019 [64]
	*Civic participation*		x	x	Phillips, 2020 [52]
	*Social gradient*	x	x	x	Persaud, 2018 [48]
	*Spirituality*		x		Velez, 2020 [49]
	*Social exclusion and inclusion*	x	x	x	Persaud, 2018 [48]
		x	x	x	Valdez, 2023 [57]
	*Stigma*	x			Daly, 2022 [61]
	*Cultural safety*	x			Daly, 2022 [61]
	*Gender and gender identity*		x		Mohammadi, 2023 [63]
			x		Phan, 2020 [50]
	*Discrimination*			x	Phillips, 2020 [52]
				x	Ogbolu, 2019 [45]
			x		Phan, 2020 [50]
		x			Killion, 2022 [55]
	*Marginalization*		x		Mohammadi, 2023 [63]
	*Social status* - immigration status		x		Mohammadi, 2023 [63]
			x		Phan, 2020 [50]
		x			De Los Santos, 2014 [43]
	*Cultural humility*		x		Velez, 2020 [49]
	*Crime and violence*	x	x		Phillips, 2020 [52]
		x			Daly, 2022 [61]
	*Diversity* - cultural diversity - cultural and ethnic diversity - race	x	x	x	Valdez, 2023 [57]
		x	x		De Los Santos, 2014 [43]
			x		Cantey, 2017 [46]
			x		Velez, 2020 [49]
			x		Mohammadi, 2023 [63]
			x		Phan, 2020 [50]
	*Equity and inequities*	x		x	Cohen, 2009 [59]
				x	De Los Santos, 2014 [43]
				x	Crawford, 2022 [56]
		x	x	x	Valdez, 2023 [57]
		x			Cantey, 2017 [46]
**Living and working conditions**	*Food insecurity and access to nutritious food* - food insecurity - access to nutritious food	x	x	x	McNeil, 2013 [60]
		x	x	x	Persaud, 2018 [48]
		x		x	Phillips, 2020 [52]
				x	Kostelanetz, 2022 [53]
			x		Mohammadi, 2023 [63]
	*Work environment*				
	*Employment status* - unemployment - job security - income - financial strain			x	Kostelanetz, 2022 [53]
				x	Daly, 2022 [61]
		x	x	x	Phillips, 2020 [52]
		x	x	x	Persaud, 2018 [48]
		x	x		Baverstock, 2018 [62]
	*Healthy/Physical environment* - environmental health (overflowing rubbish bins and filthy toilets) - environmental problems (e.g., air, water and soil)	x	x		Baverstock, 2018 [62]
			x		Mohammadi, 2023 [63]
	*Geographic isolation*	x	x	x	McNeil, 2013 [60]
	*Education and educational level*		x		Phan, 2020 [50]
		x			Phillips, 2020 [52]
	*Healthcare services* - health disparities - barriers to healthcare - access to health service - availability of health service - healthcare disparities - access to healthcare resources - quality of healthcare			x	Ogbolu, 2019 [45]
		x		x	Cantey, 2017 [46]
		x	x	x	Decker, 2017 [47]
				x	Daly, 2022 [61]
				x	Powers, 2022 [54]
		x	x		De Los Santos, 2014 [43]
		x	x		Baverstock, 2018 [62]
		x	x		Phillips, 2020 [52]
		x			Addy, 2015 [44]
		x			Cohen, 2009 [59]
	*Housing difficulties* - homelessness	x	x	x	McNeil, 2013 [60]
		x			Cohen, 2009 [59]
		x	x	x	Decker, 2017 [47]
		x			Phillips, 2020 [52]
				x	Daly, 2022 [61]
				x	Kostelanetz, 2022 [53]
		x	x		Baverstock, 2018 [62]
	*Utilities*	x	x		Phillips, 2020 [52]
	*Transportation*			x	Kostelanetz, 2022 [53]
		x	x	x	Persaud, 2018 [48]
			x		Phillips, 2020 [52]
**Social and community networks**	*Social support*		x		Phillips, 2020 [52]
		x	x	x	Persaud, 2018 [48]
	*Social connection*			x	Kostelanetz, 2022 [53]
**Individual lifestyle behaviours**	*Individual lifestyle factors* - drinking, smoking and other addictions	x	x		Baverstock, 2018 [62]
		x	x	x	McNeil, 2013 [60]
		x	x	x	Persaud, 2018 [48]
	*Health literacy* - low literacy			x	Kostelanetz, 2022 [53]
		x	x		Phillips, 2020 [52]
		x			De Los Santos, 2014 [43]
**Age, sex, constitutional factors (individual level-stable conditions)**					

## Data Availability

No new data were created in this study.

## Supplementary Materials

The following supporting information can be downloaded at: https://www.mdpi.com/article/10.3390/healthcare14050560/s1, Supplementary File S1: PRISMA Checklist; Supplementary File S2: Search strategy; Supplementary File S3: PRISMA Flowchart; Supplementary File S4: Characteristics and data extracted from the included studies.

## Abbreviations

The following abbreviations are used in this manuscript:

SDoH	Social Determinants of Health
KAP	Knowledge, Attitude, Practice
PRISMA	Preferred Reporting Items for Systematic Reviews and Meta-Analyses
PEO	Population, Exposition, Outcome
US	United States
UK	United Kingdom
COVID-19	Coronavirus Disease
