# Supplementary material for: Social Determinants of Health Assessed Among Nurses: A KAP-Oriented Systematic Review Using the Dahlgren-Whitehead Rainbow Model
Source: Healthcare (Basel). 2026 Feb 24;14(5):560. doi: 10.3390/healthcare14050560 (PMC12984389; doi:10.3390/healthcare14050560)
Supplement: Supplementary file 1 [file healthcare-14-00560-s001.zip › Supplementary File S2_Search strategy.pdf]

Supplementary File S2: search strategy

**PUBMED**	(((((((Health Knowledge, Attitudes, Practice[MeSH Terms]) OR (Attitude of Health Personnel[MeSH Terms])) OR (Health Behavior[MeSH Terms])) OR (KAP[Title/Abstract])) OR (Knowledge, Attitudes, Practice[Title/Abstract])) OR (Knowledge, Attitudes, Behavior[Title/Abstract])) AND (((((((((Nurses[MeSH Terms]) OR (Nursing[MeSH Terms])) OR(Nurs*[Title/Abstract])) OR (Students, Nursing[MeSH Terms])) OR (Student* Nurs*[Title/Abstract])) OR (Pupil Nurs*[Title/Abstract])) OR (Education, Nursing[MeSH Terms])) OR (Nurs* Education [Title/Abstract])) OR (nurs* curricul*[Title/Abstract]))) AND ((((Social Determinants of Health[MeSH Terms]) OR (Social Determinants of Health[Title/Abstract])) OR (SDoH[Title/Abstract])) OR (SDH[Title/Abstract]))
**CINHAL**	AB Health Knowledge, Attitudes, Practice OR AB Attitude of Health Personnel OR AB Health Behavior OR AB KAP OR AB Knowledge, Attitudes, Practice OR AB Knowledge, Attitudes, Behavior AND AB nurses OR AB Nursing OR AB Nurs* OR AB students, nursing OR AB Student* Nurs* OR AB Pupil Nurs* OR AB education, nursing OR AB Nurs* Education OR AB nurs* curricul*AND AB social determinants of health OR AB sdoh OR AB sdh OR TI Health Knowledge, Attitudes, Practice OR TI attitude of health personnel OR TI Health Behavior OR TI KAP OR TI Knowledge, Attitudes, Practice OR TI knowledge-attitude-behaviour AND TI Nurses OR TI Nursing OR TI Nurs* OR TI Students, Nursing OR TI Student* Nurs* OR TI Pupil Nurs* OR TI Education, Nursing OR TI Nurs* Education OR TI nurs* curricul* AND TI Social Determinants of Health OR TI sdoh OR TI sdh
**PSYCINFO**	AB Health Knowledge, Attitudes, Practice OR AB Attitude of Health Personnel OR AB Health Behavior OR AB KAP OR AB Knowledge, Attitudes, Practice OR AB Knowledge, Attitudes, Behavior AND AB nurses OR AB Nursing OR AB Nurs* OR AB students, nursing OR AB Student* Nurs* OR AB Pupil Nurs* OR AB education, nursing OR AB Nurs* Education OR AB nurs* curricul*AND AB social determinants of health OR AB sdoh OR AB sdh OR TI Health Knowledge, Attitudes, Practice OR TI attitude of health personnel OR TI Health Behavior OR TI KAP OR TI Knowledge, Attitudes, Practice OR TI knowledge-attitude-behaviour AND TI Nurses OR TI Nursing OR TI Nurs* OR TI Students, Nursing OR TI Student* Nurs* OR TI Pupil Nurs* OR TI Education, Nursing OR TI Nurs* Education OR TI nurs* curricul* AND TI Social Determinants of Health OR TI sdoh OR TI sdh
**SCOPUS**	((TITLE-ABS-KEY (health AND knowledge, AND attitudes, AND practice) OR TITLE-ABS-KEY (attitude AND of AND health AND personnel) OR TITLE-ABS-KEY (health AND behavior) OR TITLE-ABS-KEY (kap) OR TITLE-ABS-KEY (knowledge, AND attitudes, AND practice) OR TITLE-ABS-KEY (knowledge, AND attitudes, AND behavior))) AND ((TITLE-ABS-KEY (nurse) OR TITLE-ABS-KEY (nursing) OR TITLE-ABS-KEY (students, AND nursing) OR TITLE-ABS-KEY (student* AND nurs*) OR TITLE-ABS-KEY (pupil AND nurs*) OR TITLE-ABS-KEY (

	education, AND nursing) OR TITLE-ABS-KEY (nurs* AND education) OR TITLE-ABS-KEY (nurs* AND curricul*))) AND ((TITLE-ABS-KEY (social AND determinants AND of AND health) OR TITLE-ABS-KEY (sdoh) OR TITLE-ABS-KEY (sdh)))
**WEB**<br>**OF SCIENCE**	AB Health Knowledge, Attitudes, Practice OR AB Attitude of Health Personnel OR AB Health Behavior OR AB KAP OR AB Knowledge, Attitudes, Practice OR AB Knowledge, Attitudes, Behavior AND AB nurses OR AB Nursing OR AB Nurs* OR AB students, nursing OR AB Student* Nurs* OR AB Pupil Nurs* OR AB education, nursing OR AB Nurs* Education OR AB nurs* curricul*AND AB social determinants of health OR AB sdoh OR AB sdh OR TI Health Knowledge, Attitudes, Practice OR TI attitude of health personnel OR TI Health Behavior OR TI KAP OR TI Knowledge, Attitudes, Practice OR TI knowledge-attitude-behaviour AND TI Nurses OR TI Nursing OR TI Nurs* OR TI Students, Nursing OR TI Student* Nurs* OR TI Pupil Nurs* OR TI Education, Nursing OR TI Nurs* Education OR TI nurs* curricul* AND TI Social Determinants of Health OR TI sdoh OR TI sdh
