# Supplementary material for: Social Determinants of Health Assessed Among Nurses: A KAP-Oriented Systematic Review Using the Dahlgren-Whitehead Rainbow Model
Source: Healthcare (Basel). 2026 Feb 24;14(5):560. doi: 10.3390/healthcare14050560 (PMC12984389; doi:10.3390/healthcare14050560)
Supplement: Supplementary file 1 [file healthcare-14-00560-s001.zip › Supplementary File S3_PRISMA Flowchart.pdf]

Supplementary File S3: PRISMA Flowchart

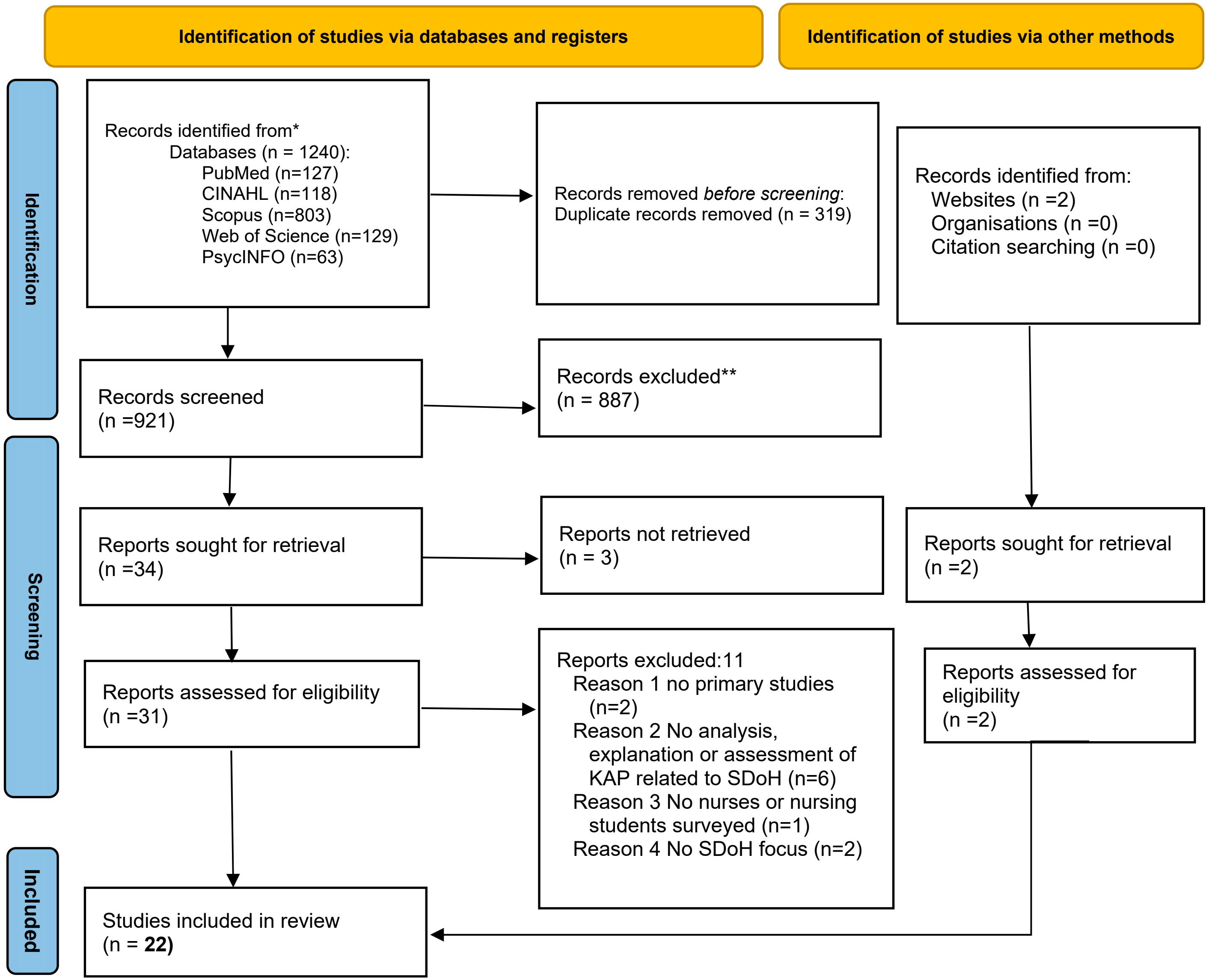

From: Page MJ, McKenzie JE, Bossuyt PM, Boutron I, Hoffmann TC, Mulrow CD, et al. The PRISMA 2020 statement: an updated guideline for reporting systematic reviews. BMJ 2021;372:n71. doi: 10.1136/bmj.n71
