# Supplementary material for: Social Determinants of Health Assessed Among Nurses: A KAP-Oriented Systematic Review Using the Dahlgren-Whitehead Rainbow Model
Source: Healthcare (Basel). 2026 Feb 24;14(5):560. doi: 10.3390/healthcare14050560 (PMC12984389; doi:10.3390/healthcare14050560)
Supplement: Supplementary file 1 [file healthcare-14-00560-s001.zip › Supplementary File S4_Characteristics and data extracted from the included studies.pdf]

Supplementary File S4: characteristics and data extracted from the included studies

Author (year)	Country	Study design	Outcome	Sample	Results	SDoH	KAP	Approaches
Cohen, (2009) [59]	Canada	Qualitative descriptive study	Explore how Canadian baccalaureate nursing programmes incorporate the development of social justice, equity and SDoH competencies into their community health clinical courses	N= 12 course leaders	Students learned about social justice, equity and SDoH. They can also gain clinical experience in nontraditional settings (e.g., Aboriginal wellness centres or homeless shelters). Their supervisors help them connect theory to practice and deepen their understanding of SDoH	Social justice, equity, SDoH	Nursing role in addressing social justice, health disparities, equity, and housing (K)<br>Use social justice and equity in clinical practice (P)	Focus group
McNeil, (2013) [60]	Canada	Qualitative	Explore clinicians' preparedness to provide SDoH-sensitive care to people experiencing homelessness, and how they overcame training gaps	N=24 doctors and nurses	Participants revealed initial unpreparedness to handle SDOH when treating homeless patients. They learned to provide care that considers and addresses social and structural factors by gaining experiential knowledge and	Addiction, housing, homelessness, food insecurity, geographic isolation	Limited understanding of SDoH (K)<br>Providing care to the homeless seems challenging (A)<br>Limited training about SDoH (P)	Interviews

					integrating it into their clinical practice.			
De Los Santos, (2014) [43]	US	Qualitative Descriptive	Describe an interprofessional, longitudinal, service-learning program for healthcare students	N= 762 (medical students, undergraduate nursing students, nurse practitioner students, undergraduate and graduate social work and law students)	Through community assessments and home visits, students learn about the impact of resource scarcity on health and well-being	Poverty, immigration status, low literacy, access to healthcare resources, cultural diversity, social justice, equity, SDoH	Impact of resource scarcity on health and well-being (K)<br>Confidence with complex health and social needs of diverse populations (A)<br>Provide services to households (P)	Interprofessional service-learning program
Addy, (2015) [44]	US	Descriptive qualitative-quantitative	Describe and evaluate an interprofessional course addressing SDoH, health disparities, patient safety, cultural competence and ethics	N= 936 (public health, social work, medicine, pharmacy and nursing students)	Students' assessment of their own knowledge about SDoH and health disparities, as a result of the course, significantly improved	SDoH, health care disparities	Health care disparities and SDoH (K)	Interprofessional Course<br>Self-reported surveys and students' reflections
Cantey, (2017) [46]	US	Qualitative	Describe a teaching	N= 7 (undergradu	The students agreed that this	Gender discrimination	Health disparities and inequities (K)	Role-played simulations

			strategy to increase the cultural awareness of nursing students using student-developed low-fidelity simulations	ate nursing students)	experience would have a positive influence on their future clinical practice. They developed a greater cultural awareness and understanding of SDoH. They also learned about specific nursing roles related to SDoH	, health behaviour, cultural and ethnic diversity	Cultural awareness in relation to cultural and ethnic diversity (A)<br>Assessing and addressing patients' needs according to their specific socioeconomic, cultural, and environmental conditions (P)	
Decker, (2017) [47]	US	Observational	Illustrate how nursing faculty have incorporated SDoH concepts using a variety of community clinical experiences throughout a pre-licensure nursing programme	N= 240 (nursing students)	Students develop caring, non-judgmental attitudes toward people living in poverty. As their autonomy and responsibility increased, they felt empowered to reduce clients' health risks	Poverty, health disparities, homeless	Understand the unique problems related to poverty (K)<br>Non-judgmental attitude towards poverty (A)<br>Address health risks (P)	Community health coursework conducted through discussions, simulations, lectures, and clinical experiences<br>Attitude Toward Poverty Scale
Baverstock, (2018) [62]	Australia	Qualitative Descriptive	Explore first-year students' understanding of the	N= 110 (pharmacy and nursing students)	In their photos and essays, 34% of students focused on social factors, 26% on	Income, housing, individual lifestyle factors (e.g.,	Students' understanding of SDoH (k)	Photograph essay assessment

			SDoH as observed by them in their environment. Identify areas for future curriculum development		individual lifestyle factors, 24% on physical determinants, 11% on the availability of health services, and 4% on policy. According to the students' essays, education and policy can contribute to health equity	alcohol and smoking), physical environment, and availability of health services	Attitude towards SDoH referred to different levels: from societal to individual ones(A)	
Persaud, (2018) [48]	US	Descriptive	Explore nurses' knowledge, attitudes, and behaviours related to SDoH	N= 107 (registered nurses)	Nurses reported varying levels of knowledge and skills about different SDoH	Income, social gradient, social exclusion, work conditions, unemploymen t and job security, social support, addiction, food insecurity, transportation , education, race and culture, and disability	High levels of knowledge and confidence in the ability to discuss social support, addiction, and transportation (K-A)<br><br>Low levels of knowledge, confidence in ability, and likelihood to discuss social gradient, food insecurity, social exclusion, unemployment, and job security (K-A-P)	SDOH survey

Ogbolu, (2019) [45]	US	Cross-sectional design	Outline research, teaching, service, and clinical practice activities implemented by nurse faculty scholars to act on the SDoH	N=57 (nurse faculty scholars)	Research field (environmental and physical stressors, service access); teaching SDoH areas (discrimination, health care quality and access for vulnerable populations); service activities (addressing discrimination); leading SDoH areas in clinical practice (health care quality and access for vulnerable populations, discrimination)	Health service access, discrimination, health disparities, quality of healthcare	Research, teaching, service activities and clinical practice (P)	Attribution of Racial/Ethnic Health Disparities Scale
Scheffer, (2019) [64]	UK, US	Before-after design	Explore the attitudes towards poverty and social justice of nursing students enrolled in UK and US courses on SDoH and health inequities	N=230 (nursing students)	US students were more likely to have positive attitudes towards social justice and poverty. They were also more likely to feel supported by their environment in acting for social justice and to intend to do so	Poverty, social justice	Positive attitude related to social justice and poverty (A)	SDoH courses<br><br>Attitudes to Poverty Scale (ATP)<br><br>Social Justice Scale (SJS)

Velez, (2020) [49]	US	Descriptive	Describe the impact of a short-term, International service-learning program in Haiti	NA*	Students developed an awareness of cultural humility and SDoH and improved their ability to adapt to different cultural and socio-economic contexts	Diversity, poverty, spirituality, cultural humility	Fostered awareness towards cultural humility and social inequalities (A)<br>Practice in complex cultural and socio-economic contexts (P)	International service-learning program
Phan, (2020) [50]	US	Pilot study with mixed methods evaluation design: post simulation debrief and post simulation survey	Evaluate the systematic implementation of SDoH simulation on students' knowledge and attitudes about the impact of SDoH on health equity. Evaluate whether the simulation is beneficial to student learning	N (simulation) = 182 N (survey)= 44 (baccalaureate nursing students)	64% of participants reported a positive change in attitude toward working with marginalised populations. 89% reported increased knowledge of the role of the nurse in addressing health equity. 75% reported increased knowledge of SDoH through the simulation	Discrimination, race, gender identity, immigration status, educational level	Nursing role in addressing SDoH and health equity (K)<br>Awareness of vulnerable populations (A)	Simulation
Kuehn, (2020) [51]	US	Before-after design with pre- and post-simulation surveys	Assessing students' attitudes towards poverty and their beliefs	N (simulation) = 41	Attitudes towards poverty improved post-simulation, with students shifting away from	Poverty	Structural explanation of poverty as a social and systemic result (A)	Community Action Poverty Simulation

			about the relationship between poverty and health	N (pre-post simulation survey)= 32 (nursing and social work students)	stigmatising and blaming individuals			The Attitudes toward Poverty-Short Form (**ATP-SF**)<br><br>Beliefs Related to Poverty and Health (**BRPH**)
Phillips, (2020) [52]	US	Cross-sectional	Assessing nurses' knowledge, self-efficacy and intended behaviours regarding integrating SDoH into clinical practice	N= 768 (nurses)	Nurses' confidence varied across the SDOH. 50% of respondents reported feeling more knowledgeable or more confident in their ability to discuss access-to-care issues with patients than with other SDoH. 40% reported not knowing how to address an identified SDOH	Access to health service, transportation, health literacy, social support, income, civic participation, crime, violence, utilities, housing, access to nutritious food, discrimination	**Good knowledge:** health service access, access to nutritious foods, health literacy, and level of education. **Poor knowledge:** utilities, income, crime, violence, housing (**K**)<br><br>**Confidence:** health service access, transportation, health literacy, and social support. **Less confidence:** income, civic participation, crime, utilities, violence (**A**)<br><br>**Less likely to talk:** civic participation, income, discrimination, utilities, access to nutritious foods (P)	71-item Social Determinants of Health Assessment Survey

Daly, (2022) [61]	Canada	Qualitative descriptive	Implementing a curriculum centred on nurse navigation to emphasise the influence of systemic processes on health and help students translate knowledge about SDoH into action	NA*	Students demonstrated the ability to identify resources and collaborate with other professionals to address client needs	Health equity, housing, stigma, violence, income, healthcare access, cultural safety	Health equity, cultural safety, stigma, trauma and violence informed care (K)<br>Competencies required to help clients access health, housing, income, and refer people to health, legal, and social services (P)	Practice course with two components: a seminar and a community-based practical component
Kostelanetz, (2022) [53]	US	Mixed-Methods	Investigate attitudes, facilitators, barriers, and perceived roles in the implementation of universal SDoH screening as a routine part of patient care	N= 193 (social workers, case managers, pharmacists, physicians, nurses, advanced practice providers, administrative)	Participants argue that information about patients' social needs could be used to improve patient care, communication and trust with patients. Barriers to SDoH screening are: lacking resources to address identified needs, time to ask, support staff to ask, and training in responding to identified needs	Housing, financial strain, food insecurity, health literacy, transportation needs, social connection	Positive attitude towards universal screening for SDoH (A)<br>Lack of training to respond to social needs (P)	Online survey, interviews

Powers, (2022) [54]	US	Descriptive qualitative	Examine the experiences and perspectives of health professions students who participated in a six-month interprofessional student hotspotting program	N= 24 (health professions students: medicine, social work, pharmacy, nursing, health psychology)	The participants learned to value a patient-centred, empathic approach to care and to address social as well as medical needs	Poverty and barriers to healthcare	Identification and management of SDoH and the ability to visualise their impact on patients' health status (P)	Interprofessional program<br>Focus group
Killion, (2022) [55]	US	Qualitative	Describe the development and implementation of a poverty simulation, reflect on the process, and present the reactions of the participants involved	N=29 (nursing, public health, medicine students, community healthcare and social service providers)	Participants gained an inside view of poverty	Poverty, discrimination	Daily challenges, discrimination and lack of opportunities (K)	Role-playing case studies
Crawford, (2022) [56]	US	Qualitative-quantitative approach	Examine the impact of a social justice course on graduate nursing students' values and attitudes	N= 41 (Doctor of Nursing Practice students)	The course enhanced attitudes, perceived control, and intentions, impacting students personally,	Social justice, equity	Increased knowledge and understanding of social justice(K)<br>Positive attitudes towards social justice values, goals and behaviours (A)	Course designed on a multidisciplinary model

			toward social justice		professionally, and academically		Increased sensitivity to social injustice and inequality, and confidence in the ability to achieve social justice (P)	Social justice scale (SJS)
Valdez, (2023) [57]	US	cross-sectional exploratory	Obtain data about associate degree in nursing faculty knowledge, skills and attitudes about diversity, equity and inclusion in nursing	N=318 (associate degree in nursing)	As knowledge increases, skills tend to increase as well. As knowledge increases, positive attitudes tend to increase. As skills increase, attitudes tend to increase	Diversity, equity, inclusion	Participants report having knowledge (K) and skills (P) to address diversity, equity, and inclusion in practice	Diversity, Equity, and Inclusion (DEI) survey
Mohammadi, (2023) [63]	Iran	Descriptive	Determine the awareness and attitudes of students and professors toward the social determinants of health	NA* (Professors and students)	Professors were more aware of the SDoH than students, and their attitudes were less positive than those of students	Environmental problems, health equity, gender, race, marginalisation, poverty, social status, food insecurity	SDoH (A)	Questionnaire about awareness and attitude towards SDoH
Holland, (2024) [58]	US	Descriptive Qualitative	Explore the experience of baccalaureate nursing students	N=56 (baccalaureate nursing students)	Five themes emerged: “emotions”, “personal history of poverty”,	Poverty	Understanding about SDoH issues and their role in driving change (K)	Community Action Poverty Simulation

			participating in a Community Action Poverty Simulation and its impact on empathy and social justice awareness		"empathy", "rising advocacy", "lessons learned		Awareness of SDoH, holistic perspective of the injustices caused by poverty (A)	
							Development of interpersonal and social empathy (P)	

Explanations: * unreported exact sample size; (K) Knowledge; (A) Attitudes; (P) Practice.
